# Investigating the APOE4‐associated heterogeneity of imaging biomarkers across AD diagnostic stages

**DOI:** 10.1002/alz.087489

**Published:** 2025-01-09

**Authors:** Jason Mares, Anurag Sharma, Jia Guo, Tal Nuriel

**Affiliations:** ^1^ Columbia University, New York, NY USA; ^2^ Columbia University Irving Medical Center, New York, NY USA; ^3^ Zuckerman Institute, Columbia University, New York, NY USA; ^4^ Department of Cell Biology and Pathology, New York, NY USA; ^5^ Taub Institute for Research on Alzheimer's disease, New York, NY USA

## Abstract

**Background:**

Carriers of the apolipoprotein E ε4 (APOE4) allele are at an increased risk of developing Alzheimer’s disease (AD) and display an earlier age of onset compared to non‐carriers who develop the disease. While researchers have extensively characterized the impact of APOE4 on the susceptibility of AD, little is known about the differences in disease etiology and presentation in APOE4 carriers vs. non‐carriers.

**Method:**

In order to investigate these differences, we performed a broad analysis of imaging biomarkers in APOE4 carriers vs. non‐carriers present in the ADNI cohort.

**Result:**

We observed differences between APOE4 carriers vs. non‐carriers in several imaging readouts, including Ab‐PET, Tau‐PET, FDG‐PET, and structural MRI measures. These differences were variable based on sex and amyloid positivity. Interestingly, when we compared cognitively normal ADNI participants who converted to MCI or AD to those who did not, we observed APOE4‐dependent differences that may point to specific pathologies that mediate MCI/AD conversion in APOE4 carriers vs. non‐carriers.

**Conclusion:**

These observations of pathological heterogeneity between APOE4 carriers vs. non‐carriers reveal significant differences in disease pathogenesis between these two groups, which may have important implications with regard to the diagnosis and treatment of AD in different genetic cohorts.

